# Alteration of circulating platelet-related and diabetes-related microRNAs in individuals with type 2 diabetes mellitus: a stepwise hypoglycaemic clamp study

**DOI:** 10.1186/s12933-022-01517-5

**Published:** 2022-05-20

**Authors:** Ceren Eyileten, Zofia Wicik, Disha Keshwani, Faisal Aziz, Felix Aberer, Peter N. Pferschy, Norbert J. Tripolt, Caren Sourij, Barbara Prietl, Florian Prüller, Dirk von Lewinski, Salvatore De Rosa, Jolanta M. Siller-Matula, Marek Postula, Harald Sourij

**Affiliations:** 1 Department of Experimental and Clinical Pharmacology, Center for Preclinical Research and Technology CEPT, Medical University of Warsaw, Banacha 1B str., 02-097 Warsaw, Poland; 2 Genomics Core Facility, Center of New Technologies (CeNT), University of Warsaw, Warsaw, Poland; 3 Division of Endocrinology and Diabetology, Interdisciplinary Metabolic Medicine Trials Unit, Medical University of Graz, Graz, Austria; 4 Center for Biomarker Research in Medicine, CBmed, Graz, Austria; 5 Division of Cardiology, Department of Internal Medicine, Medical University of Graz, Graz, Austria; 6 Clinical Institute of Medical and Chemical Laboratory Diagnostics, Medical University of Graz, Graz, Austria; 7 Division of Cardiology, Department of Medical and Surgical Sciences, “Magna Graecia” University, Catanzaro, Italy; 8 Department of Cardiology, Medical University of Vienna, Vienna, Austria

**Keywords:** miRNA, Platelets, Platelet reactivity, Bioinformatic analysis, miRNA-gene target interaction, Network, Biomarker, Diagnosis, Prognosis

## Abstract

**Background:**

In patients with type 2 diabetes mellitus (T2DM) an association between severe hypoglycaemic episodes and the risk of cardiovascular (CV) morbidity and mortality has been previously established.

**Methods:**

We aimed to investigate the influence of hypoglycaemia on several diabetes-related and platelet-related miRNAs selected based on bioinformatic analysis and literature search, including hsa-miR-16, hsa-miR-34a, hsa-miR-129-2, hsa-miR-15a, hsa-miR-15b, hsa-miR-106a, miR-223, miR-126. Selected miRNAs were validated by qRT-PCR in 14 patients with T2DM on metformin monotherapy, without established CV disease and antiplatelet therapy during a stepwise hypoglycaemic clamp experiment and a follow-up 7 days after the clamp event. In order to identify which pathways and phenotypes are associated with validated miRNAs we performed target prediction on genes expressed with high confidence in platelets.

**Results:**

Circulating levels of miR-106a-5p, miR-15b, miR-15a, miR-16-5p, miR-223 and miR-126 were increased after euglycaemic clamp followed by hypoglycaemic clamp, each with its distinctive time trend. On the contrary, miR-129-2-3p, miR-92a-3p and miR-34a-3p remained unchanged. MiR-16-5p was negatively correlated with interleukin (IL)-6, intercellular adhesion molecule (ICAM) and vascular cell adhesion molecule (VCAM) (p = 0.002, p < 0.001, p = 0.016, respectively), whereas miR-126 was positively correlated with VCAM (p < 0.001). There were negative correlations between miR-16-5p, miR-126 and coagulation factors, including factor VIII and von Willebrand factor (vWF). Among all studied miRNAs, miR-126, miR-129-2-3p and miR-15b showed correlation with platelet function. Bioinformatic analysis of platelet-related targets of analyzed miRNAs showed strong enrichment of IL-2 signaling. We also observed significant enrichment of pathways and diseases related to cancer, CV diseases, hyperglycemia, and neurological diseases.

**Conclusions:**

Hypoglycaemia can significantly influence the expression of platelet-enriched miRNAs, with a time trend paralleling the time course of platelet activation. This suggests miRNAs could be exploited as biomarkers for platelet activation in response to hypoglycaemia, as they are probably released by platelets upon activation by hypoglycaemic episodes. Should they hold their promise in clinical endpoint studies, platelet-derived miRNAs might become helpful markers of CV risk in subjects with diabetes.

*Trial registration* The study was registered at clinical trials.gov; Impact of Hypoglycaemia in Patients With DIAbetes Mellitus Type 2 on PLATElet Activation (Diaplate), trial number: NCT03460899

## Introduction

Since patients with type 2 diabetes mellitus (T2DM) are several times more likely to develop cardiovascular (CV) complications compared to those without diabetes, multifactorial CV risk factor control including glycemic control is crucial in preventing CV events [1]. Hypoglycaemia is one potential side effect of sulfonylurea and insulin treatment. There is an association between severe hypoglycaemic episodes (i.e. hypoglycaemia requiring third party assistance) and risk of CV morbidity and mortality has been shown in patients with T2DM. Hypoglycaemia increases the risk of CV complications as it increases pro-atherothrombotic factors [2]. Moreover, hormones release due to hypoglycaemia induces cardiac arrhythmias and increases workload, which correlates with a higher risk of atherothrombosis. Additionally, hypoglycaemia induces platelet activation and makes the coagulation cascade more potent [3]. Hyperinsulinaemic hypoglycaemia clamp studies indicated deranged oxidative stress and inflammation parameters 24 h following the hypoglycaemic episode in T2DM subjects [4]. However, it was previously described that fibrinolytic processes that are mediated by altered levels of coagulation factors might be impaired for up to 1 week following hypoglycaemia [5]. Aberer et al*.* [3] demonstrated a sustained effect of hypoglycaemia on platelet function, coagulation, fibrinolysis, and endothelial function up to 7 days after hypoglycaemic clamp. Increased platelet activation in response to acute hypoglycaemia may be the result of the release of counter-regulatory catecholamines [6]. Moreover, it may also predispose platelet death by disrupting calcium homeostasis and mitochondrial function and integrity [7]. Several biochemical disturbances have been observed following acute- and repeated-hypoglycaemia including increased levels of plasminogen activator inhibitor-1 (PAI-1), increased thrombin generation and increased plasma levels of clotting factor VIII [8].

Platelets are found to be a significant source of miRNAs, small (18–25 nucleotides), non-coding RNAs, that influence various aspects of cellular functions through the regulation of gene expression. A single miRNA can influence the expression of multiple genes, and one single target can be regulated by several miRNAs [9, 10]. Circulating miRNAs can be detected in many body fluids including plasma, serum, or whole blood, and they are stable in biological samples. Thus, they can be used as diagnostic and prognostic novel biomarkers as well as novel therapeutic targets [11, 12]. Due to the number of limitations of currently used platelet function tests, circulating miRNAs could be promising biomarkers of inflammation and platelet function and were previously described to participate in processes of platelet activation and aggregation [9, 13]. Alteration in their expression is associated with various biological processes, including platelet reactivity, glucose metabolism, antiplatelet drug response, and anti-inflammatory response [9]. The most studied miRNAs related to platelet reactivity, namely miR-223 and miR-126 were found to be altered in CV diseases (CVDs) patients and correlated with the antiplatelet treatment response [14, 15]. However, to date, no studies aimed to analyze the miRNA expression related to platelets in response to acute hypoglycaemia in T2DM patients.

In the current study, we have selected analyzed miRNAs based on bioinformatic analysis including the top platelet-related miRNAs (hsa-miR-16, hsa-miR-34a, hsa-miR-129-2, hsa-miR-15a, hsa-miR-15b, hsa-miR-106a) [10]. Moreover, we analyzed both miR-223 and miR-126, as they were one of the most studied miRNAs and were shown to be abundantly present in platelets and involved in platelet reactivity [9, 16, 17]. Consequently, we aimed to investigate the influence of hypoglycaemia on several selected miRNAs in subjects at an early stage of T2DM on metformin monotherapy, without additional established CVD and antiplatelet therapy during a stepwise hypoglycaemic clamp experiment and a follow-up 7 days after the clamp event.

## Methods

### Study group

The study design, both inclusion and exclusion criteria, and study population were previously described in detail [3]. Briefly, 14 patients diagnosed with T2DM according to American Diabetes Association criteria [18] aged 40 to 63 years were included in the study; all participants were on a stable metformin monotherapy for a period of at least 90 days prior to screening or diet only. Body mass index (BMI) was between 20.0 and 34.0 kg/m^2^ (both inclusive) and a glycated haemoglobin (HbA1c) concentration between 43 and 64 mmol/mol (6.0–8.0%; both inclusive). All participants underwent a hyperinsulinaemic-euglycaemic and subsequently a hyperinsulinaemic-hypoglycaemia clamp experiment as previously described [3]. Both clamp investigations were performed at the Clinical Trials Unit at the Medical University of Graz, Austria, after a 10-h overnight fasting period. Samples for platelet activation and coagulation markers as well as values for markers of endothelial function, inflammation and counterregulatory hormones were obtained 30 min after reaching the designated plateau (3.5 mmol/L and 2.5 mmol/L, respectively of blood glucose) as well as after recovery from hypoglycaemia at a plasmaAQ1 glucose level of 5.5 mmol/L, 1-day and 7-days thereafter..

### Blood collection and biomarker measurement

For the current analyses we used samples collected prior to the euglycaemic clamp, prior to the hypoglycaemic clamp as well as those collected 1-day and 7-days after the hypoglycaemia clamp experiment. At all those time points also platelet activity, coagulation, endothelial function and inflammatory markers were determined [3].

### RNA preparation, detection, and quantification of miRNAs by quantitative PCR

The Maxwell RSC miRNA from Tissue and Plasma or Serum isolation kits (Promega Corporation) was used for total RNA extraction using 100 μL of plasma aliquots. Promega Maxwell RSC 48 Modular Automated Nucleic Acid Preparation System was used for total RNA extraction. Plasma was preprocessed with Proteinase K and Lysis Buffer in the volumes described within the manufacturers’ protocols. The mixture was placed on a vortex mixer at 3000 rpm for 5 s, and then left at 37 °C for 15 min. After transferring prepared lysate to the Maxwell RSC Cartridge DNase I Solution was added. Total RNA was eluted by 30 μL of nuclease-free water (Applied Biosystems, CA). The RNA concentration was measured using Qubit RNA high sensitivity (Invitrogen). Subsequently, the obtained RNA template was subjected to a reverse transcription reaction using the TaqMan miRNA Reverse Transcription kit (ABI, California, USA) according to guidelines provided by the manufacturer. Afterwards, miRNA expressions were detected by quantitative polymerase chain reaction (qPCR) using TaqMan miRNA Assay kits (ABI, California, USA) for the corresponding miRNAs on a CFX384 Touch Real-Time PCR Detection System (BioRad Inc. Hercules, California, USA). Cel-miR-39 was spiked-in as an exogenous normalizer. Mean values of all reactions—performed in triplicate—were used in statistical analysis as previously described [19, 20]. MiRNA expressions were expressed as 2−ΔCT [19, 21, 22].

### Statistical analysis

All results for categorical variables were presented as a number and percentage (%). Continuous variables were expressed as mean ± standard deviation (SD) or median and interquartile range (IQR), depending on the normality of distribution assessed by the Shapiro–Wilk test. Wilcoxon sign-rank test for paired two groups and One-Way ANOVA Tukey HSD test were applied for multiple groups comparison. A linear mixed-effects model was applied to assess the longitudinal associations of inflammatory, endothelial, coagulation, and platelet markers with miRNAs. Study participants were included as a random intercept and study visits were included as a random slope in the random effects component of the model. While study visits and inflammatory, endothelial, coagulation, and platelet markers were included as fixed effects in the fixed effect component of the model. All tests were two-sided, and p-value < 0.05 was considered statistically significant. Median and 95% confidence interval of log10-transformed miRNA expressions were presented by box-plots. Statistical analyses were performed in SPSS version 22.0 (IBM Corporation, Chicago, USA) and Stata version 16.1 (Stata Corp, Houston, TX, USA).

### Ethical considerations

The study was approved by the Ethics Committee of the Medical University of Graz, Austria, (Ethics Committee number: EK30-012 ex 17/18 for the main study and 33-515 ex 20/21 for the current analysis) and a written consent, for all study-related procedures, was given by all study participants. The study was conducted in accordance with the Declaration of Helsinki, as well as the guidelines laid down by the International Conference on Harmonization for Good Clinical Practice (ICH GCP E6 guidelines). Recruitment of the participants was performed with the use of a local recruitment registry (Graz Diabetes Registry for Biomarker Research, GIRO). The study was registered at clinical trials.gov (https://clinicaltrials.gov/ct2/show/NCT03460899│NCT03460899).

### Bioinformatics analysis miRNA targets prediction, data filtering, and visualization as interaction networks

#### Platelet-related tissue specific expression

To identify potentially targeted genes by selected miRNAs which are expressed in platelets and/or plasma we mined Tissues 2.0 database [23]. We selected genes showing at least 1 confidence of expression in blood platelets and plasma. The median expression confidence level for platelets was 0.917 according to the database (0-min., 5-max.). We identified 3194 genes for this expression confidence cut-off in analyzed tissues.

#### Target predictions

To identify targets of analyzed miRNAs (hsa-miR-106a.5p,hsa-miR-126-3p, hsa-miR-126-5p, hsa-miR-15a-5p, hsa-miR-15b-3p, hsa.miR-15b-5p, hsa-miR-16-5p, hsa-miR-223-3p, hsa-miR-223-5p, hsa-miR-129-2-3p) we used the multiMiR 1.4 R package [24]. We screened all conserved target sites in 14 target prediction databases. The top 20% predicted target sites of each external database were queried.

#### Enrichment analyses

Enrichment analyses of Reactome signaling pathways were performed using the Reactome R package [25, 26]. Enrichment analysis of signaling pathways was performed using EnrichR database API plugin and BioPlanet_2019 database, analysis of diseases was performed using Jensen_DISEASES datasets. In all analyses, the false discovery rate adjusted p-value cutoff was set as lower than 0.05. In order to identify signaling cascades related to impaired platelet activity we performed enrichment analysis screening of the BioPlanet_2019 pathway database. Simultaneously in order to identify the disease traits which would be helpful in precise identification of the risk groups associated with platelet dysfunctions, we performed enrichment analysis of Jensen Diseases databases. For further analyses we selected the top ten most significant pathways and diseases for each analyzed miRNA.

## Results

### Participants

Samples taken from all fourteen participants (10 men and four women, age 55 ± 7 years, BMI 28.9 ± 3.3 kg/m^2^) were available for miRNAs evaluation and included in the statistical analysis. Detailed baseline characteristics and demographic data are summarized in Table 1.

**Table 1:** Participant’s demographics

Parameter	N = 14				
Male sex (%)	10 (71.4%)				
Age (years)	55 ± 6.5				
BMI (kg/m^2^)	28.9 ± 3.3				
Weight (kg)	86.4 ± 15.1				
SBP (mmHg)	133 ± 13				
DBP (mmHg)	83 ± 8				
Fasting plasma glucose (mmol/L)	7.2 ± 0.90				
Triglycerides (mmol/L)	2.02 ± 1.33				
Cholesterol (mmol/L)	5.22 ± 1.19				
HDL cholesterol (mmol/L)	1.19 ± 0.36				
LDL cholesterol (mmol/L)	3.13 ± 1.01				
Diabetes duration (years)	5 ± 4				
Daily metformin dose (mg)	1336 ± 599				
HbA1c, mmol/mol (%)	51 ± 7 (6.8 ± 2.8)				
ACE inhibitors, n (%)	4 (28.6)				
Angiotensin-II receptor antagonists, n (%)	5 (35.7)				
Calcium antagonists, n (%)	3 (21.4)				
Diuretics, n (%)	1 (7.1)				
Statins, n (%)	4 (28.6)				

miRNAs expressions					
miRNAs	Before euglycaemic clamp	Before hypoglycaemic clamp	1-day after hypoglycaemic	7-day after hypoglycaemic	P value
miR-106a-5p	5.56 [5.14–6.79]	5.51 [4.81–7.69]	6.61 [5.97–7.69]	7.95 [6.40–8.19]	0.126
miR-15b	5.77 [5.32–6.90]	5.48 [5.28–8.94]	8.03 [7.04–8.64]	8.13 [6.67–8.85]	**0.026**
miR-15a	7.27 [7.07–7.63]	7.25 [6.45–7.63]	7.49 [7.39–7.72]	7.93 [7.21–8.11]	**0.009**
miR-16-5p	7.17 [6.71–7.52]	6.94 [6.36–7.85]	7.52 [7.43–7.71]	7.65 [7.23–8.34]	**0.016**
miR-223	7.32 [6.70–7.58]	6.99 [6.43–7.32]	7.74 [7.41–8.11]	8.12 [7.80–8.60]	**0.002**
miR-126	6.48 [5.90–6.66]	6.46 [5.96–6.91]	6.80 [6.56–7.78]	7.09 [6.59–7.64]	**0.003**
miR-129–2-3p	5.53 [5.41–5.65]	5.35 [5.14–5.54]	5.38 [5.27–5.58]	5.47 [5.22–5.60]	0.789
miR-92a-3p	5.90 [5.61–6.15]	5.91 [5.65–6.20]	5.73 [5.59–6.01]	5.91 [5.70–6.11]	0.473
miR-34a-5p	5.37 [5.11–5.55]	5.18 [4.82–5.38]	5.18 [5.18–6.47]	5.42 [5.31–6.21]	0.443

Data are presented as mean ± SD and median and IQR based on the data distribution. Multiple groups comparison p value was calculated based on One-way ANOVA Tukey test

*ACE* angiotensin-converting-enzyme, *BMI* body mass index, *SBP* systolic blood pressure, *DBP* diastolic blood pressure, *HbA1c* glycated haemoglobin, *HDL* high density lipoprotein, *LDL* low density lipoprotein

Bold p value indicates statistical significance < 0.05

### Alteration of circulating miRNAs

**Fig. 1:**
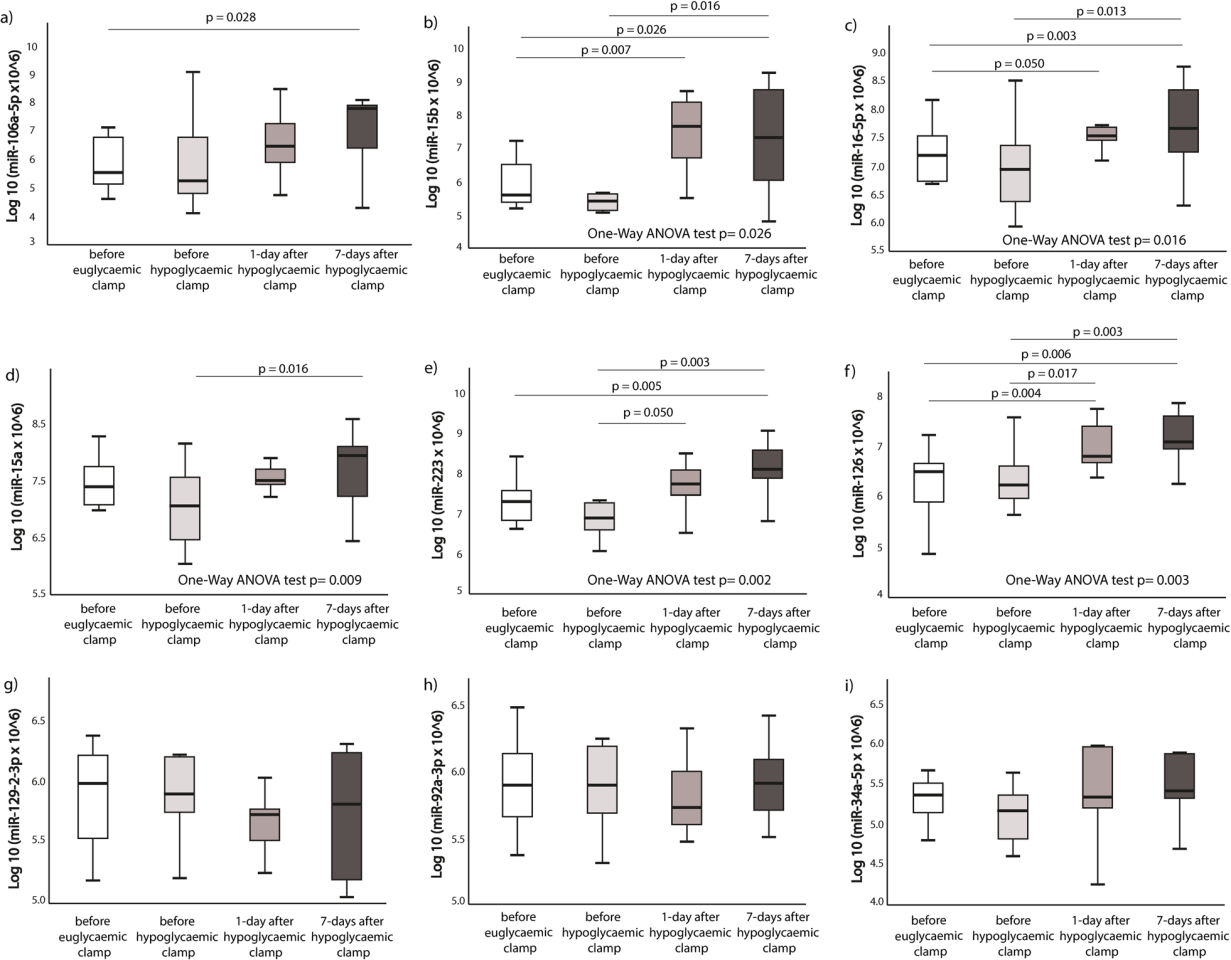
Effect of hypoglycaemia on miRNAs expression levels. **a** miR-106a-5p; **b** miR-15b; **c** miR-16-5p; **d** miR-15a; **e** miR-223; **f** miR-126; **g** miR-129–2-3p; **h** miR-92a-3p; **i** miR-34a-5p. Two groups comparison p value was calculated by Wilcoxon paired test, multiple groups comparison was calculated based on One-way ANOVA. MiRNAs expression data was presented as log10 transformation. Only statistical significant p values between the groups are shown (i.e. p ≤ 0.05)

MiR-15b, miR-15a, miR-16-5p, miR-223 and miR-126 showed increasing trend after euglycaemic clamp followed by hypoglycaemic clamp, whereas miR-129-2-3p, miR-92a-3p and miR-34a-3p remained unchanged. MiR-15b, miR-16-5p, miR-15a, miR-223 and miR-126 expression levels differed among the 4 groups analyzed by One-Way ANOVA test (p = 0.026, p = 0.016, p = 0.009, p = 0.002 and p = 0.003, respectively). There was no statistical significance between before hypoglycaemic clamp and euglycaemic clamp in none of the studied miRNAs (see Fig. 1).

### Correlations between miRNAs expression inflammatory, coagulation markers and platelet function

MiR-16-5p was negatively correlated with interleukin (IL)-6, intercellular adhesion molecule (ICAM) and vascular cell adhesion molecule (VCAM) (p = 0.002, p < 0.001, p = 0.016, respectively), whereas miR-126 was positively correlated with VCAM (p < 0.001). There were negative correlations between miR-16-5p, miR-126 and coagulation factors, including factor VIII and von Willebrand factor (vWF). Among all studied miRNAs, miR-126, miR-129-2-3p and miR-15b showed correlation with platelet function (see Table 2).

**Table 2:** MiRNA expression relation with platelet function, coagulation and inflammation markers

miRNAs	Parameter	Coef.	95% CI	*p*
miR-16-5p	Factor VIII (%)	− 0.021	− 0.038, − 0.003	**0.019**
	vWF-activity (%)	− 0.014	− 0.026, − 0.001	**0.030**
	IL-6 (pg/mL)	− 0.459	− 0.747, − 0.172	**0.002**
	ICAM (ng/mL)	− 0.007	− 0.011, − 0.003	**< 0.001**
	VCAM (ng/mL)	− 0.004	− 0.007, − 0.001	**0.016**
miR-126-5p	Factor VIII (%)	− 0.017	− 0.033, − 0.001	**0.034**
	vWF-activity (%)	− 0.013	− 0.024, − 0.001	**0.028**
	CD62^pos^ (%)	− 0.230	− 0.420, − 0.041	**0.017**
	VCAM (ng/mL)	0.005	0.003, 0.008	**< 0.001**
miR-34a-5p	PAI-1 (ng/mL)	0.073	0.012, 0.133	**0.018**
miR-129-2-3p	PAI-1 (ng/mL)	− 0.035	− 0.067, − 0.002	**0.037**
	PAC1^pos^CD62^pos^CD63^pos^ (%)	− 16.295	− 30.733, − 1.857	**0.027**
miR-15b	PAC1^pos^CD62^pos^CD63^pos^ (%)	56.935	11.719, 102.152	**0.014**

*miRNA* miR, microRNA, *CI* confidence interval, *CD62* P-selectin, *vWF* von Willebrand Factor, *IL-6* interleukin-6, *ICAM* intercellular adhesion molecule, *VCAM* vascular cell adhesion protein, *PAI-1* plasminogen activator inhibitor-1, *PAC1* activated GP IIb/IIIa

Bold value indicate that only statistically significant correlations between the parameters are shown (i.e. p<0.05)

### Bioinformatic analysis results

#### Identification of the pathways affected by selected miRNAs

**Fig. 2:**
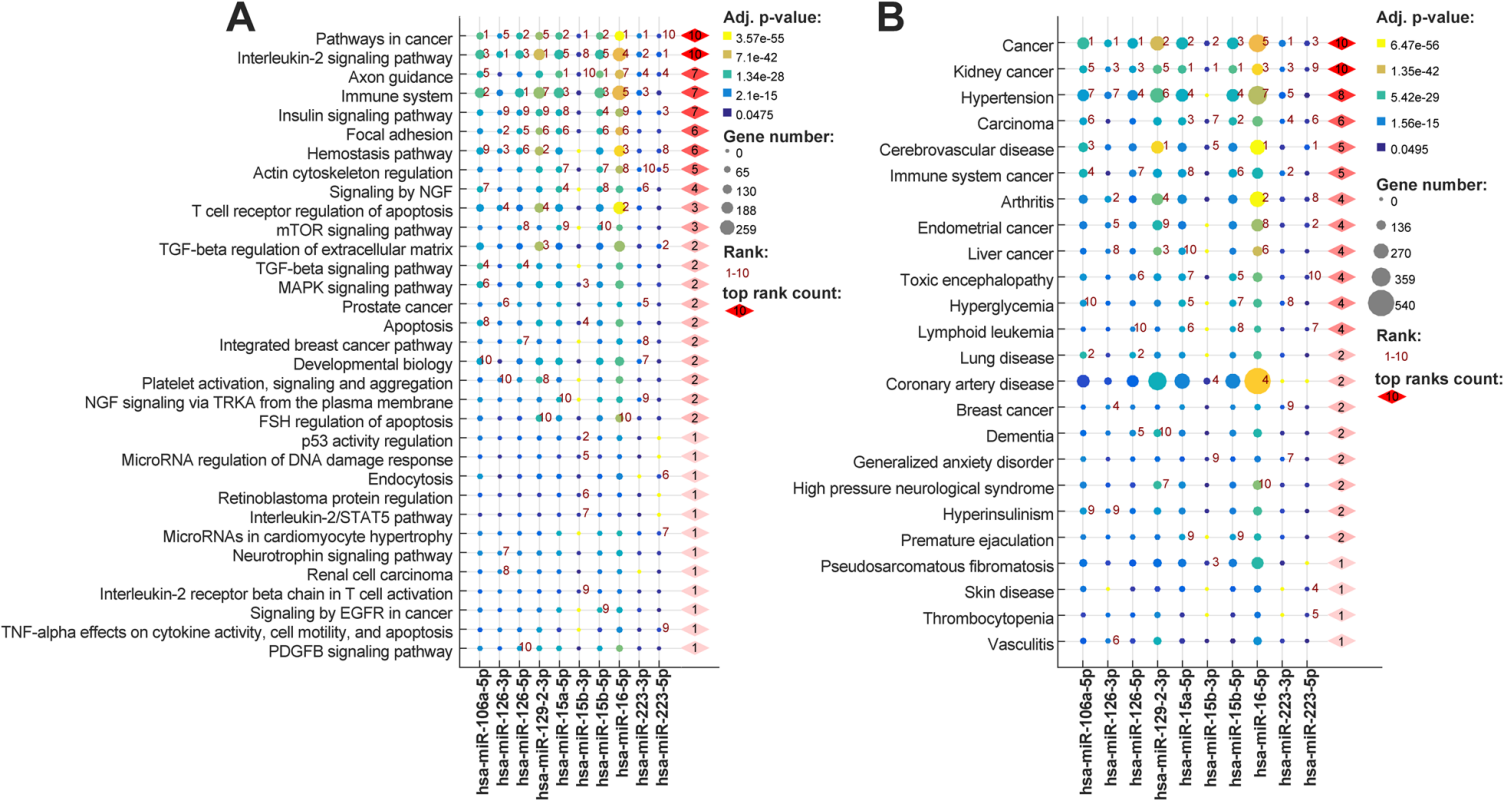
Top ten Pathways (**A**) and Diseases (**B**) associated with targets expressed in platelets of each validated miRNAs. Ontological terms were selected based on adjusted p value, and ordered by the increasing summary rank order (top ranks count)

In order to identify which pathways are associated with validated miRNAs we performed target prediction on genes expressed with high confidence in platelets (see Fig. 2A). We performed separate enrichment analyses for following miRNAs: hsa-miR-106a-5p, hsa-miR-126 (-3p and -5p), hsa-miR-15a-5p, hsa-miR-15b (-3p and -5p), hsa-miR-16-5p, hsa-miR-223 (-3p and -5p) and hsa-miR-129-2-3p selecting top most significant ontological terms for each of them. Our analysis identified pathways in cancer and IL-2 signaling pathways as the most significantly affected by the all analyzed miRNAs. For pathways in cancer hsa-miR-106a-5p, hsa-miR-15b-3p, hsa-miR-16-5p and hsa-miR-223-3p had the lowest adjusted p values. While the IL-2 signaling pathway was the strongest associated with the targets of hsa-miR-126-3p, hsa-miR-129-2-3p, hsa-miR-223-5p. Other most affected pathways were axon guidance (7 miRNAs, most significant for hsa-miR-15a-5p, hsa-miR-15b-5p), immune system (7 miRNAs, most significant for hsa-miR-126-5p), insulin signaling pathway (7 miRNAs), focal adhesion (6 miRNAs), hemostasis pathway (6 miRNAs). Among pathways strictly related to platelets we observed enrichment of platelet activation, signaling and aggregation (hsa-miR-126-3p, hsa-miR-129-2-3p).

#### Identification of the diseases phenotypes associated with selected miRNAs

Disease enrichment analysis showed that targets present in platelets of all analyzed miRNAs are associated with cancer related phenotypes (including kidney cancer and carcinoma phenotypes) (see Fig. 2B). We also observed a high number of miRNAs associated with hypertension (9 miRNAs, most significant for hsa-miR-129-2-3p, hsa-miR-223-5p), cerebrovascular disease (8 miRNAs), arthritis (7 miRNAs, most significant for hsa-miR-126-3p). Other enriched phenotypes present among top 10 hits included hyperglycemia (4 miRNAs), coronary artery disease (3 miRNAs), dementia (2 miRNAs), high pressure neurological syndrome (1 miRNA), hyperinsulinism (1 miRNA) and thrombocytopenia (1 miRNA).

## Discussion

Up to this day, only a few studies have directly investigated the impact of hypoglycaemia on thrombotic parameters, platelet function, and inflammatory biomarkers. In the first stage, applying bioinformatics analysis tools, we associated crucial biological function and signaling pathways related to the miRNAs with the most prominent differential expression pattern in T2DM associated with glucose metabolism, inflammation, thrombosis, and platelet function. Our experimental stage showed for the first-time sustained changes of several miRNAs expression, including miR-15a, miR-15b, miR-16, miR-223, miR-126 up to 7 days after hypoglycaemia induced by a hyperinsulinaemic-hypoglycaemic clamp. Importantly, the observed expression of miRNAs during the experiment presented a similar pattern of changes as biomarkers of platelet activation described previously by our group [3]. Moreover, our bioinformatic analysis showed the pathways associated with the platelet-related targets of validated miRNAs identified pathways in cancer and IL-2 signaling as those most affected by all of the validated miRNAs. Enrichment analysis of the diseases identified cancer-related terms, including kidney cancer and carcinoma phenotypes, as most significantly associated with those miRNAs. We also observed significant enrichment of pathways and diseases related to CVDs, hyperglycemia, and neurological diseases.

MiR-15b, among other miRNAs, was identified in our latest bioinformatic analysis [10] as regulating the highest number of platelet-related genes and highest number of regulatory targets. However, data on its relationship with platelet function are conflicting. Costa de Freitas et al*.* proved that miR-15b-5p is downregulated in platelets in patients with acute coronary syndrome (ACS) and high platelet reactivity (HPR) and clopidogrel modulates miR-15b-5p expression levels in vitro [27]. MiR-15b protects against HPR in patients undergoing percutaneous coronary intervention through B-cell lymphoma 2 (Bcl-2)-mediated platelet apoptosis [28]. Nonetheless, similarly to the current observation, its expression was positively associated with platelet reactivity in non-ST elevation ACS patients in different time-points in both cohorts included in the study [29]. It was suggested that miR-15b-5p modulates platelet reactivity by targeting SUMO Specific Peptidase 5 (*SENP5)*, a SUMO isopeptidase that is activated in the context of platelet activation, ischemia–reperfusion injury and cardiomyopathy [30, 31]. Additionally, high levels of plasma miR-15b-5p were significantly associated with transforming growth factor (TGF)β1/2 expression and higher risk of venous thromboembolism recurrence [32].

MiR-126 may influence platelet reactivity on many levels, including induction by collagen and regulation of platelet’s surface receptor expression including CD62 (P-selectin) [14]. In patients with T2DM, miR-126 expression correlated with platelet activation measured by soluble P-selectin and responded with increasing doses of acetylsalicylic acid [33]. Recently it was described that miR-126 might be associated with CV events through modulation of platelet-mediated thrombin generation [14, 34]. MiR-126 is highly expressed in endothelial cells and platelets and plays a pivotal role in vascular diseases and angiogenesis [16, 17, 21, 35]. It is therefore possible that lower expression of P-selectin associated with higher expression levels of miR-126-5p in our study has been implicated in protective properties by suppressing NOTCH receptor 1 (*Notch1*) homolog and reducing thrombogenicity in T2DM by targeting platelet tissue factor [43]. It suggests that high expression of miR-126-5p can be protective against HPR [36]. Similar results were also observed in ACS patients included in the TRILOGY-ACS cohort [29]. Our data showed that induced hypoglycaemia might increase miR-126-5p expression that persists up to 7 days after hypoglycaemic clamp. In patients with type 1 diabetes and healthy individuals hypoglycaemia increases concentrations of several circulating molecules, including PAI-1, vascular endothelial growth factor (VEGF), VCAM, ICAM, E-selectin, P-selectin and IL-6 [37]. Our published results showed that VCAM and ICAM increased gradually, reaching a peak at day-7 after the hypoglycaemic clamp experiment in T2DM patients [3]. Importantly, previously it was shown that miR-126-3p directly inhibits the expression of VCAM-1 [38], and it regulates the expression of VCAM-1 in several clinical conditions, including atherosclerosis [39, 40]. In line with previous findings on negative correlation with CD62-P-selectin, we found a positive correlation between miR-126-5p expression and VCAM-1 level. It should be noted that miR-126 also regulates endothelial cell adhesion and vascular inflammation by decreasing the adhesion of leukocytes to endothelial cells by binding to VCAM-1 [41]. Similar to these findings, we found a negative correlation between miR-126 and vWF activity and factor VIII levels. It was previously shown that vWF activity represents endothelial dysfunction and is along with factor VIII are significantly increased after hypoglycaemia [37]. It was also demonstrated that endothelial microparticles derived from endothelial cells exposed to high glucose concentration contained lower amounts of miR-126, potentially impairing endothelial repair capacity [42]. Similarly, it may be hypothesized that increased miR-126-5p expression in response to hypoglycaemia may act as a counterregulatory and protective anti-inflammatory mechanism as up-regulated expression of VCAM-1 exacerbates inflammatory cells’ infiltration, which was observed in our previous study. Therefore, upregulation of miR-126-5p may play a protective role from glucose-induced dysfunction.

Similarly, we found overexpression of miR-16 at day-7 after hypoglycaemic clamp. MiR-16 is predicted to target several genes, including downstream genes encoding proteins involved in insulin signaling. As a result, miR-16 might impact insulin resistance and as a result inhibit cell apoptosis induced by hyperglycemia [43]. It was previously demonstrated that the expression levels of miR-16 were reduced in hyperglycaemia. However, no data supports our observation on the impact of acute hypoglycaemia on miR-16 expression. Animal studies showed that miR-16-5p is upregulated during calorie-restricted diet and modifies inflammatory cytokines released from macrophages [44]. Hypoglycaemia increases pro-inflammatory cytokines, but the underlying mechanisms, including miRNAs expressions and the relationship with counterregulatory physiological responses are not completely understood [45]. Our previous study showed that the IL-6 level is significantly increased during the hypoglycaemic clamp and reaches a peak at the end of the clamp. However, we could not observe an increase at 24 h or 7 days after hypoglycaemia. Surprisingly, VCAM and ICAM did not change during the hypoglycaemic clamp experiment but increased after that, reaching a peak at day-7 [3]. In the current study, we found that increased expression of miR-16 is negatively correlated with inflammatory biomarkers, including IL-6, VCAM-1, and ICAM-1. In vitro and in vivo animal studies described that miR-16 may be related to atherosclerosis and coronary artery disease by participating in the inflammatory process [46]. MiR-16 was associated with suppressed atherosclerotic plaque formation and decreased accumulation of proinflammatory factors including IL-6, tumor necrosis factor (TNF)-α, monocyte chemoattractant protein-1 (MCP-1), and IL-1β but increased secretion of anti-inflammatory factors like IL-10 and TGF-β [46]. Anti-inflammatory mechanisms of miR-16 are complex including downregulation of several targets like nuclear factor-κB (NF-κB) or NOD-like receptor protein 3 (NLRP3) inflammasome [47]. Animal studies confirmed the involvement of miR-16 in the regulation of vascular remodeling in response to hypoperfusion [48]. Another well described target of miR-16 is toll-like receptor 4 (*TLR4*) and its expression is suppressed by miR-16 [47]. Activation of TLRs can trigger signaling cascades and stimulate production of several inflammatory cytokines. Based on our results, one could speculate that an increased expression of the anti-inflammatory miR-16 in response to acute hypoglycaemia might provide some protection against the harmful effects of subsequent hypoglycaemia as it is negatively correlated with inflammatory biomarkers. It should be noted that upregulation of miR-16 expression was also described in patients with acute myocardial infarction [49]. It was also found that miR-16 along with miR-15a correlate with adverse events at 12-month follow-up in T2DM patients who have undergone percutaneous intervention to correct critical limb ischemia [50]. MiR-15a and miR-16 play essential roles in cell apoptosis, including platelets, regulating apoptotic signaling pathways. Recent data have shown miR-15a and miR-16 suppress angiogenesis by targeting VEGF and fibroblast growth factor (FGF)-2 [51]. Further studies are needed to determine the long-term consequences of acute and repeated hypoglycaemia on miRNAs expression, their inflammatory function, and their involvement in atherogenesis and their prognostic values.

Moreover, our bioinformatic analysis identified IL-2 signaling pathway which was the strongest associated with the targets of hsa-miR-126-3p, hsa-miR-129-2-3p, hsa-miR-223-5p. IL-2 association with glucose metabolism is not well characterized. It suggested that IL-2 and IL-6 cytokines by regulating the dynamics of host response may concurrently contribute to alterations in the glucose level [52]. Previous study showed that contrasting with the hyperglycaemic effects of the acute phase mediator IL-6, the T-cell cytokine IL-2 seems to support glucose uptake and utilization by immune cells [52]. Literature data also shows that IL-2 reverses established type 1 diabetes in non-obese diabetic mice by a local effect on pancreatic regulatory T cells [53]. On the other hand, high doses of IL-2 has been found to produce durable antitumor responses, most benefiting patients with melanoma and renal cell carcinoma [54]. These results are convergent with our bioinformatic analysis which showed enrichment of cancer-related phenotypes including carcinoma and kidney cancer and pointing out association between analyzed miRNAs, platelet activity, IL-2 pathway and cancerogenesis. Interestingly, analysis of the gene components of enriched signaling pathways identified overlap between the platelet receptors PAC1 (KLF6), CD62-SELP and CD63 analyzed in this study, enriched pathways and diseases. PAC1 (KLF6) showed overlap with the highly significant IL-2 signaling pathway and TGF-beta signaling pathway. Association of CD62-SELP was observed in significant pathways: hemostasis pathway, platelet activation, signaling and aggregation, TNF-a effects on cytokine activity, cell motility, and apoptosis. For diseases, it was kidney cancer, hypertension, carcinoma, cerebrovascular disease, arthritis, coronary artery disease, thrombocytopenia, vasculitis. Presence of CD63 was observed in hemostasis pathway and platelet activation, signaling and aggregation. Our bioinformatic analysis results suggest that the IL-2 signaling pathway could be an interesting therapeutic target in the context of hypoglycaemic episodes and the risk of CV morbidity.

Taken together, our experimental stage showed that hypoglycaemia might influence several miRNAs expressions, including miR-15a, miR-15b, miR-16, miR-223, and miR-126, importantly we confirmed their indirect association with platelet function assays. As most of the analyzed miRNAs are highly abundant in platelets, based on patterns of expression changes that mimics patterns of realizing platelet activation biomarkers one can speculate that they are released upon their activation in response to hypoglycaemic episodes. The observed dynamics of changes in miRNA expression seem to confirm the results of our previous study [3], which showed a delayed and persistent process of platelet activation in response to hypoglycaemia. Importantly, we also found in bioinformatic analysis that hypoglycaemia may induce inflammatory processes that may play a role in atherogenesis and negatively affect prognosis.

## Limitations

The study’s major limitation is that only a fraction of all known miRNAs related to processes influenced by hypoglycaemia were included in the analysis. However, our primary goal was to evaluate miRNAs known to be related to platelet reactivity. Therefore, the miRNAs analyzed in the present study were chosen based on our previous publications and bioinformatic analysis focusing on platelet-related miRNAs [55]. Other miRNAs might also alter platelet reactivity, inflammation, or thrombosis induced by hypoglycaemia. Secondly, we used samples collected from a limited cohort of T2DM individuals included in a mechanistic study that aimed to investigate the effect of hypoglycaemia on platelet and coagulation activation biomarkers. Another limitation of the study is reduced interpretability of the results due to lack of targets-related expression, and thus limited possibility to tell if enriched pathways and diseases were activated or inhibited. Solution for this will be planned for future large scale analysis of the non-coding RNAs together with coding transcripts.

## Conclusions

Our study found hypoglycaemia to significantly influence the expression of platelet-enriched miRNAs, with a time trend paralleling the time course of platelet activation. This suggests they could be exploited as biomarkers for platelet activation in response to hypoglycaemia, as they are probably released by platelets upon activation by hypoglycaemic episodes. We believe that platelet-derived miRNAs in the context of T2DM associated with established CV risk factors may help to improve monitoring the short and long-term risk of CV events. The involvement of platelets and platelet’s related miRNAs in many processes leading to CV complications in T2DM provides a promising area for research and potential clinical application. Further studies in bigger cohorts are needed to determine the long-term consequences of acute and repeated hypoglycaemia on the expression of miRNAs related to platelet function, thrombosis and inflammation, and their involvement in atherogenesis and thus prognosis in this population.

## Data Availability

The datasets used and/or analyzed during the current study are available from the corresponding author on reasonable request.

## Abbreviations

ACE: Angiotensin-converting-enzyme
ACS: Acute coronary syndrome
Bcl-2: B-cell lymphoma 2
BMI: Body mass index
CV: Cardiovascular
CVD: Cardiovascular disease
CI: Confidence interval
CD62: P-selectin
DBP: Diastolic blood pressure
ERK1/2: Extracellular signal-regulated kinase 1/2
FGF: Fibroblast growth factor
HbA1c: Glycated haemoglobin
HDL: High density lipoprotein
HPR: High platelet reactivity
IL: Interleukin
ICAM: Intercellular adhesion molecule
IQR: Interquartile range
LDL: Low density lipoprotein
MAPK: Mitogen-activated protein kinase
MCP-1: Monocyte chemoattractant protein-1
MiRNA: MiR: microRNA
NF-κB: Nuclear factor-κB
Notch1: Notch receptor 1
NLRP3: NOD-like receptor protein 3
PAI-1: Plasminogen activator inhibitor-1
PAC1: Activated GP IIb/IIIa
PLXNB2: Plexin B2
PI3K/AKT: Phosphatidylinositol 3‑kinase/protein kinase B
qPCR: Quantitative polymerase chain reaction
SBP: Systolic blood pressure
SD: Standard deviation
SENP5: SUMO Specific Peptidase 5
T2DM: Type 2 diabetes mellitus
TLR4: Toll-like receptor 4
TNF: Tumor necrosis factor
TGF: Transforming growth factor
VCAM: Vascular cell adhesion molecule
VEGF: Vascular endothelial growth factor
vWF: Von Willebrand factor
